# Clinical utility of using C-reactive protein and procalcitonin as biomarkers for a novel neonatal sepsis diagnostic platform (ASCMicroPlat)

**DOI:** 10.1186/cc11793

**Published:** 2012-11-14

**Authors:** K McAllister, M Sheridan-Pereira, N O'Sullivan, R O'Kelly, D Mark, G Czilwik, C Martin, O Sheils, J O'Leary

**Affiliations:** 1 Trinity College Dublin, Ireland; 2 The Coombe Women and Infants University Hospital, Dublin, Ireland; 3 HSG-IMIT, Lab-on-a-Chip, Freiburg, Germany

## Background

Neonatal sepsis contributes significantly to morbidity and mortality and encompasses a multisystemic inflammatory response to a microorganism. A lack of suitable diagnostic biomarkers and delays in microbe identification (48 to 72 hours) challenge clinical decision-making. C-reactive protein (CRP) is the current biomarker for sepsis in patients; however, use of earlier markers such as procalcitonin (PCT) could prove more effective. This study aims to assess the clinical utility of combining CRP and PCT for a novel sepsis diagnostic biomarker platform (ASCMicroPlat).

## Methods

Blood samples were obtained from 350 infants evaluated for sepsis at the Coombe Women and Infants University Hospital. Eligible infants met one or more inclusion criteria: age ≤6 weeks, clinical sepsis signs, and/or maternal sepsis risk factors. The puncture site was sterilized with alcohol prior to phlebotomy for microbial and biomarker tests. Blood was collected in clotting activator tubes, and incubated at room temperature for 1 hour before centrifuging to separate out serum for later acute-phase protein quantification. For gold-standard tests, an immunoturbimetric CRP assay was performed using a Beckman analyser and a chemiluminescent Elecsys BRAHMS PCT immunoassay using a Cobas e602 analyser.

## Results

The mean age of infant evaluation was 2.29 ± 10.37 days. The average gestational age at birth was 37.04 weeks (4.27), 30% of which were preterm. Eighteen out of 350 samples (5%) were blood culture positive. Thirteen samples met the criteria for a laboratory-confirmed bloodstream infection (LCBSI), the most common organisms being *Escherichia coli *(28%) and *Streptococcus agalactiae *(22%). CRP results (*n *= 350) ranged from ≤0.1 to 226 mg/l, sepsis-positive (≥5 mg/l) CRP tests occurred in 69% of LCBSI. PCT quantified samples to date (*n *= 100) ranged from 0.03 to 67.23 ng/ml. Both analytes featured in higher cases that suggested sepsis than positive blood culture numbers: 56 out of 350 cases had CRP levels ≥5 mg/l, for PCT 23 out of 100 individuals tested positive at the cutoff (>0.5 ng/ml) for bacterial infection.

## Conclusion

Sepsis diagnosis requires accurate detection biomarkers; preliminary study findings show that CRP is present in the majority of bloodstream infections. Furthermore, results using both biomarkers suggest that blood culture results do not indicate all cases of true sepsis. Further research will correlate the completed CRP and PCT gold-standard dataset with that produced by ASCMicroPlat PCR-based assays to assess the clinical utility of this device for sepsis diagnostics.

